# A School for Health Officers

**Published:** 1901-12

**Authors:** 


### TEXAS MEDICAL JOURNAL.

AUSTIN, TEXAS.

A MONTHLY JOURNAL OF MEDICINE AND SURGERY.

EDITED AND PUBLISHED BY
F. E. DANIEL, M. D.

ASSOCIATE EDITOR:
WITTEN BOOTH RUSS, M. D.,
San Antonio, Texas.

Published Monthly at Austin, Texas. Subscription price $1.00 a year in advance.

Eastern Representative: John Guy Monihan, St. Paul Building, 220 Broadway, New York City.

Official organ of the State Association of Health Officers, the West Texas Medical Association, the Houston District Medical Association, the Austin District Medical Society, the Brazos Valley Medical Association, the Galveston County Medical Society, and several others.

### A SCHOOL FOR HEALTH OFFICERS.

Under the above caption our friend Atkinson, long time secretary American Medical Association, now editor of *Public Health*, Philadelphia, has an excellent article from which we extract a few sentences below. We take the subject for our text in connection with the approaching meeting (January 15, 1902, at Austin) of the Texas State Association of Health Officers. The State Association of Health Officers has a great work before it, the nature and extent of which, I feel assured, is not sufficiently known and appreciated even by its members. The situation in Texas is peculiar. Our quarantine laws take cognizance of nothing except imported diseases. There is no provision whatever for the institution of sanitary reforms throughout the State looking to the prevention of those diseases of local origin, and due to filth, seepage water, defective plumbing, want of drainage, etc., and which carry off annually more people than all the epidemics of yellow fever and smallpox combined. True, the law does provide for the appointment by the county judge in each county of a county health officer, and the health officer of each county is supposed to be a part of the health machinery of the State. But his services are utilized by the State Health Officer only in connection with smallpox or yellow fever (cholera and bubonic plague are out of consideration in this connection). Even then he is not paid by the State, and there is no provision in the law for paying him, and no penalty for non-performance of duty. It is true he is made subordinate to the State Health Officer, and may obey him, or not, just as he likes. Moreover, there is no way to make a judge appoint a county health officer, and in some counties none are appointed. When one is appointed—by the judge—(what does a judge know about a doctor's fitness for such duties?) it is too often the case that the appointment is made from personal or political considerations; and hence, we have in Texas some county health officers—in name only, I am sorry to say. I was much tempted to express it in more emphatic if less elegant language.

Under the circumstances, the health officers in Texas must become a law to themselves—those who are earnest, intelligent, and who do not mind a little self-sacrifice for the welfare of their people. The Association of Health Officers in Texas,—which meets semi-annually,—should be made a school in which the older and more experienced sanitarians, with the State Health Officer in the lead, should instruct them as to their duties and how best they can secure the reforms in their several communities so necessary to stay the ravages of typhoid fever, malaria, dysentery, diphtheria, scarlet fever,—and consumption. They can hardly hope for any help from the State. They must take the initiative, and enlighten their people, beginning with the county judges and the county commissioners; begin a campaign of sanitary education. Personally, I have advised several county health officers—friends of mine—to get an occasional mass meeting of the leading citizens and read the riot act to them, tell them that the cause of the typhoid fever which is almost perennially endemic in some communities is to be found in the abominable drinking water, mostly from shallow wells—seep water; the abominable, stinking privies the aroma of which burdens the atmosphere of many a lovely little village and makes a man anxious to get away, if business or what not calls him there for a few hours or a day;—tell them that their soil is damp; that drainage will rid them of the dangers of malaria, and lots of things; that the one most pressing sanitary reform needed is drainage, sewers, and fresh and pure drinking water. We cannot, in this limited space, embrace the entire curriculum that ought to be taught in a school for health officers, but will say that every town of one thousand or more inhabitants should have a system of sewers and water works, and that if they are not able to build them there is plenty of capital available for the purpose. The delegation of rich men from New York who looked over the situation in Texas last spring are aware of this need, and their sanitary member, Dr. Soper, with whom I had several interviews, has, in his report, recommended that this very field offers inducement for safe and remunerative investment. In towns where there can be no sewers, and where the old surface privy must still be used, the sanitary officers (city physicians) should get the council to enforce the cleansing of the receptacles at short stated periods under heavy penalty.

I hope to see at the January meeting (Austin) a large attendance of health officers. President Jones has prepared an address on needed sanitary reforms or something like, and it will be worth hearing.

Dr. Atkinson (*Public Health*) says:

School for Health Officers.—Vermont provides a most practical method by which to prepare its health officers for the best performance of their duties. A school for health officers was held in Burlington, July, 1901, which was attended by some 150 officers. The expenses of attendance are paid by the State, thus insuring a good attendance.

It was shown that since the first meeting of this kind a great improvement has taken place in the health of the State, unquestionably due to the great efficiency of the local boards. Scarlet fever has declined in number of deaths over 55 per cent. Diphtheria and its allies, 50 per cent.; consumption, 31 per cent.; whooping cough the same; typhoid fever, 63 per cent.; measles increased 21 per cent., etc.

The great need of sanitary laws was clearly shown. The State has a right to enforce such laws even to interference with personal and property rights. Every man owes a duty to society not to imperil the life and health of others. Life and health are the most important objects of legislation, and the laws that govern them cast an obligation on all members of society.

A paper on the duties and responsibilities of health officers showed that not every one was fitted for this position. Training is needed—a special education. This officer must know much of law, medicine, practical physics, engineering and human nature. He must face all emergencies, know what to do, and dare to perform.

Good water supplies were needed, each town should have its water examined at a State laboratory, and when the fault was ascertained, it should at once be remedied. Typhoid fever is constantly occurring from faults in the water supplies.

It was stated that while sanitary laws must be in accord with public sentiment, in this particular Vermont is among the first. No other State has a free laboratory, nor has invested so high powers in its State board. It was suggested that the College of Vermont should grant a degree in medical sanitation. The State needs an institution for free treatment of tuberculosis.

[So does Texas.—Ed.]

